# Recurrent Antibiotic-Treated Pyelonephritis Depletes Renal Functional Reserve Despite Preserved Basal GFR in a Reflux-Prone Mouse Model

**DOI:** 10.1681/ASN.0000001166

**Published:** 2026-06-05

**Authors:** Evan A. Rajadhyaksha, Samuel Arregui, Vijay Saxena, Alleé Shaw, Jennifer L. Anderson, Sayeh Gholampoor, Gokul Achayaraj, Rose Odom, Danielle E. Soranno, David S. Hains, Andrew L. Schwaderer

**Affiliations:** 1 Division of Nephrology, Department of Pediatrics, Indiana University, Bloomington, Indiana; 2 School of Medicine, Medical Student Program for Research and Scholarship Program, Indiana University, Bloomington, Indiana

**Keywords:** fibrosis, GFR, pediatric nephrology, pyelonephritis, renal fibrosis, renal function decline, vesicoureteral reflux (VUR), basic science

## Introduction

Vesicoureteral reflux (VUR) predisposes children to recurrent pyelonephritis and subsequent risk of long-term kidney injury, with outcomes classically linked to dimercaptosuccinic acid (DMSA)–detected cortical scarring. Contemporary clinical data suggest that recurrent febrile urinary tract infection (UTI) can be accompanied by declining kidney function, tied to number of lifetime UTI events, and not associated with DMSA scars.^1^ We hypothesized that recurrent antibiotic-treated pyelonephritis produces diffuse fibrotic remodeling and that renal functional reserve can serve as a dynamic “stress-test” to identify subclinical kidney injury.

## Methods

Female C3H/HeOuJ mice with VUR underwent transurethral inoculation with uropathogenic *Escherichia coli* (CFT073) to model pyelonephritis^2^ and were treated with cefpirome^3^ beginning at 48 hours postinoculation. Mice received zero (control), one (single), or four (recurrent) infection–treatment cycles at 1-week intervals. Basal and stimulated measured GFR^4^ (mGFR) values were recorded and renal functional reserve, calculated as % change between basal and stimulated mGFR, was assessed at baseline and 2 months after final infection. Fibrosis was quantified histologically using Masson's trichrome stain. See Supplemental Methods for a full experimental timeline (Supplemental Figure 1) and additional information pertaining to the Methods.

## Results

**Figure 1:**
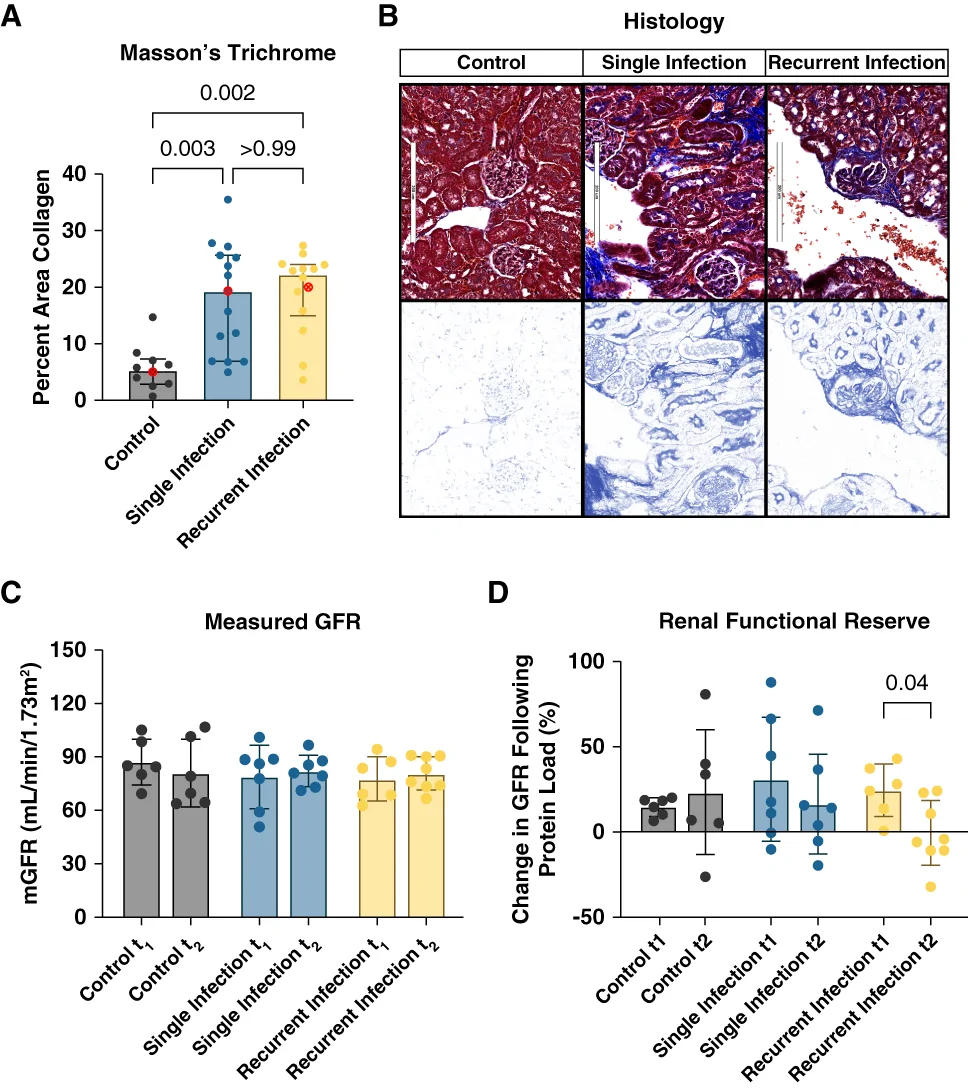
**Fibrosis, kidney function, and renal functional reserve postrecurrent urinary tract infections.** (A) Quantification of percent area collagen by Masson's trichrome. Bars show the median of each group; scale bars show interquartile range. A representative sample from each group is marked with a red dot and displayed on histology in (B). (B) Row 1 contains representative brightfield histology images of Masson's trichrome stain for each group. Row 2 contains blue collagen enrichment isolated from trichrome using color deconvolution. (C) Unstimulated mGFR for cohort 2, comparing preintervention (t1) to postintervention, at least 8 weeks after final infection, (t2) values. Bars represent means, and scale bars are SD for (C) and (D). (D) Renal functional reserve (% change in GFR from baseline when stimulated with enteral protein load) measurement for each group. The meaning of t1 and t2 remains the same. Statistical comparison on the graph represents paired *t* test from t1 to t2. mGFR, measured GFR.

Both single and recurrent pyelonephritis episodes increased collagen deposition compared with controls by Masson's trichrome stain (Figure 1, A and B). An antibiotic-only control subgroup did not differ from untreated controls, supporting that the observed collagen signal reflected infection-related remodeling rather than cephalosporin exposure. In contrast to basal mGFR, which remained stable over time and similar across all groups (Figure 1C), renal functional reserve was eliminated only in the recurrent infection group (mean change of approximately −27% from beginning to end of study). Renal functional reserve was preserved in the noninfected control and single infection groups.

## Discussion

In this refluxing mouse model, both single and recurrent episodes of antibiotic-treated pyelonephritis led to measurable fibrotic remodeling. However, this structural footprint did not translate into reduced steady-state postinfectious glomerular filtration, as basal mGFR was preserved in all groups. In contrast to basal mGFR, a significant decline in renal functional reserve from baseline to the end of the study was seen only in the recurrent infection group. This finding reframes the postinfectious consequences of pyelonephritis beyond the traditional scarring paradigm that dominates VUR outcome assessment. Specifically, kidneys maintain steady-state filtration while losing the capacity to augment GFR under physiologic demand. Loss of measured renal functional reserve with preserved basal GFR may demonstrate chronic ongoing recruitment of residual function at baseline and increased single nephron GFR, and this pattern is observed early in hyperfiltrating conditions like diabetic kidney disease.^5^

The existing body of literature in pyelonephritis and VUR focuses on scarring as the primary outcome measure. In most models, antibiotic initiation is delayed at least 5 days postinfection to maximize scarring.^6^ We started antibiotic treatment 48 hours into the infection course, and the scarring outcomes were milder than what is typically seen in the literature. This mirrors clinical observations in which delayed antibiotic treatment is a risk factor for DMSA-detected scarring. We believe that through early initiation of antibiotics, we have captured an underreported phenotype of experimental pyelonephritis in VUR, with mild scarring but a decline in the functional outcome of renal functional reserve after recurrent infection. Clinically, what we observe may correspond to an underinvestigated clinical population of children with UTI and VUR who lack scarring on DMSA—children who may have diminished renal functional reserve but whose GFR will not decline until early adulthood.

We address potential limitations of this study: First, the spacing of infection cycles 1 week apart left a short 72-hour interval between completion of antibiotics and subsequent inoculation. Altering the duration of interepisode intervals could yield different histologic trajectories or strengthen differential fibrosis between single and recurrent infection groups. Second, heterogeneity in infection severity could also have contributed to variability in results. We validated our infection and treatment methodology with urine cultures and kidney and bladder bacterial burdens at the 24-hour and 7-day time points, demonstrating infection and resolution of infection, but future work may also include immunostaining (*e.g*., CD45 and macrophage/*T*-cell markers) to link inflammatory injury to functional outcomes. Third, renal functional reserve in mice is an emerging approach; although modeled after established protein-loading paradigms, protocol optimization and external validation are needed. Fourth, approximately 10% of GFR measurements required technical repeat within 1 week; although repeat frequency did not differ by group, standardized repeat timing and sensitivity analyses remain important.

In conclusion, our findings align with and extend prior murine work demonstrating that antibiotic-treated pyelonephritis leads to renal scar formation. To add to this work, we highlight renal functional reserve as a complementary functional end point that may capture early injury not reflected by basal GFR alone. These results support the concept that reliance on DMSA scarring and basal eGFR may not capture all children who have functional consequences from pyelonephritis. These data support a shift from structural end points toward integrating dynamic functional testing when modeling infection-related kidney injury in VUR, and renal functional reserve may prove to be a useful tool in translational studies assessing reflux nephropathy and recurrent pyelonephritis.

## Supplementary Material

## Data Availability

Original data generated for the study are available in a public access repository. Data Type: Raw Data/Source Data. Repository Name: Figshare. https://doi.org/10.6084/m9.figshare.31224355.

## Supplemental Material

Supplemental Methods.

Supplemental Figure 1. Experimental timeline.
