# Supplementary material for: Recurrent Antibiotic-Treated Pyelonephritis Depletes Renal Functional Reserve Despite Preserved Basal GFR in a Reflux-Prone Mouse Model
Source: J Am Soc Nephrol. 2026 Jun 5;37(9):2027–9. doi: 10.1681/ASN.0000001166 (PMC13567891; doi:10.1681/ASN.0000001166)
Supplement: Supplementary file 1 [file jasn-37-2027-s001.pdf]

ASN American Society of Nephrology JASN Journal of the American Society of Nephrology KIDNEY360 Accessing Our World From Every Angle CJASN Clinical Journal of the American Society of Nephrology

ASN Journal Disclosure Form

As per ASN journal policy, I have disclosed any financial relationships or commitments I have held in the past 36 months as included below. I have listed my Current Employer below to indicate there is a relationship requiring disclosure. If no relationship exists, my Current Employer is not listed.

G. Achayaraj reports the following:
Employer: Indiana University School of Medicine; and Ownership Interest: Tesla, Inc. publicly traded stock; unrelated to the submitted work; ETF(TQQQ).

I understand that the information above will be published within the journal article, if accepted, and that failure to comply and/or to accurately and completely report the potential financial conflicts of interest could lead to the following: 1) Prior to publication, article rejection, or 2) Post-publication, sanctions ranging from, but not limited to, issuing a correction, reporting the inaccurate information to the authors' institution, banning authors from submitting work to ASN journals for varying lengths of time, and/or retraction of the published work.

Name: Gokul Achayaraj
Manuscript ID: JASN-2026-000163R1
Manuscript Title: Recurrent Pyelonephritis Causes Kidney Fibrosis and Impairs Renal Functional Reserve Without Impacting Basal GFR
Date of Completion: May 7, 2026
Disclosure Updated Date: May 7, 2026

ASN
American Society of Nephrology
JASN
Journal of the American Society of Nephrology
KIDNEY360
Accessing Our World From Every Angle
CJASN
Clinical Journal of the American Society of Nephrology

ASN Journal Disclosure Form

As per ASN journal policy, I have disclosed any financial relationships or commitments I have held in the past 36 months as included below. I have listed my Current Employer below to indicate there is a relationship requiring disclosure. If no relationship exists, my Current Employer is not listed.

J. Anderson has nothing to disclose.

I understand that the information above will be published within the journal article, if accepted, and that failure to comply and/or to accurately and completely report the potential financial conflicts of interest could lead to the following: 1) Prior to publication, article rejection, or 2) Post-publication, sanctions ranging from, but not limited to, issuing a correction, reporting the inaccurate information to the authors' institution, banning authors from submitting work to ASN journals for varying lengths of time, and/or retraction of the published work.

Name: Jennifer Lynne Anderson
Manuscript ID: JASN-2026-000163R1
Manuscript Title: Recurrent Pyelonephritis Causes Kidney Fibrosis and Impairs Renal Functional Reserve without Affecting Basal GFR
Date of Completion: May 22, 2026
Disclosure Updated Date: May 22, 2026

ASN American Society of Nephrology
JASN Journal of the American Society of Nephrology
KIDNEY360 Accessing Our World From Every Angle
CJASN Clinical Journal of the American Society of Nephrology

ASN Journal Disclosure Form

As per ASN journal policy, I have disclosed any financial relationships or commitments I have held in the past 36 months as included below. I have listed my Current Employer below to indicate there is a relationship requiring disclosure. If no relationship exists, my Current Employer is not listed.

S. Arregui reports the following:
Employer: IU School of Medicine

I understand that the information above will be published within the journal article, if accepted, and that failure to comply and/or to accurately and completely report the potential financial conflicts of interest could lead to the following: 1) Prior to publication, article rejection, or 2) Post-publication, sanctions ranging from, but not limited to, issuing a correction, reporting the inaccurate information to the authors' institution, banning authors from submitting work to ASN journals for varying lengths of time, and/or retraction of the published work.

Name: Samuel Arregui
Manuscript ID: JASN-2026-000163R1
Manuscript Title: Recurrent Pyelonephritis Causes Kidney Fibrosis and Impairs Renal Functional Reserve Without Impacting Basal GFR
Date of Completion: May 8, 2026
Disclosure Updated Date: May 8, 2026

ASN American Society of Nephrology | JASN Journal of the American Society of Nephrology | KIDNEY360 Accessing Our World From Every Angle | CJASN Clinical Journal of the American Society of Nephrology

ASN Journal Disclosure Form

As per ASN journal policy, I have disclosed any financial relationships or commitments I have held in the past 36 months as included below. I have listed my Current Employer below to indicate there is a relationship requiring disclosure. If no relationship exists, my Current Employer is not listed.

S. Gholampoor has nothing to disclose.

I understand that the information above will be published within the journal article, if accepted, and that failure to comply and/or to accurately and completely report the potential financial conflicts of interest could lead to the following: 1) Prior to publication, article rejection, or 2) Post-publication, sanctions ranging from, but not limited to, issuing a correction, reporting the inaccurate information to the authors' institution, banning authors from submitting work to ASN journals for varying lengths of time, and/or retraction of the published work.

Name: Sayeh Gholampoor
Manuscript ID: JASN-2026-000163R1
Manuscript Title: Recurrent Pyelonephritis Causes Kidney Fibrosis and Impairs Renal Functional Reserve Without Impacting Basal GFR
Date of Completion: May 18, 2026
Disclosure Updated Date: May 15, 2026

ASN American Society of Nephrology JASN Journal of the American Society of Nephrology KIDNEY360 Accessing Our World From Every Angle CJASN Clinical Journal of the American Society of Nephrology

ASN Journal Disclosure Form

As per ASN journal policy, I have disclosed any financial relationships or commitments I have held in the past 36 months as included below. I have listed my Current Employer below to indicate there is a relationship requiring disclosure. If no relationship exists, my Current Employer is not listed.

D. Hains has nothing to disclose.

I understand that the information above will be published within the journal article, if accepted, and that failure to comply and/or to accurately and completely report the potential financial conflicts of interest could lead to the following: 1) Prior to publication, article rejection, or 2) Post-publication, sanctions ranging from, but not limited to, issuing a correction, reporting the inaccurate information to the authors' institution, banning authors from submitting work to ASN journals for varying lengths of time, and/or retraction of the published work.

Name: David S. Hains
Manuscript ID: JASN-2026-000163R1
Manuscript Title: Recurrent Pyelonephritis Causes Kidney Fibrosis and Impairs Renal Functional Reserve Without Impacting Basal GFR
Date of Completion: May 6, 2026
Disclosure Updated Date: May 6, 2026

ASN
American Society of Nephrology
JASN
Journal of the American Society of Nephrology
KIDNEY360
Accessing Our World From Every Angle
CJASN
Clinical Journal of the American Society of Nephrology

ASN Journal Disclosure Form

As per ASN journal policy, I have disclosed any financial relationships or commitments I have held in the past 36 months as included below. I have listed my Current Employer below to indicate there is a relationship requiring disclosure. If no relationship exists, my Current Employer is not listed.

R. Odom reports the following:
Employer: Eli Lilly and Company

I understand that the information above will be published within the journal article, if accepted, and that failure to comply and/or to accurately and completely report the potential financial conflicts of interest could lead to the following: 1) Prior to publication, article rejection, or 2) Post-publication, sanctions ranging from, but not limited to, issuing a correction, reporting the inaccurate information to the authors' institution, banning authors from submitting work to ASN journals for varying lengths of time, and/or retraction of the published work.

Name: Rose Odom
Manuscript ID: JASN-2026-00163R1
Manuscript Title: Recurrent Pyelonephritis Causes Kidney Fibrosis and Impairs Renal Functional Reserve Without Impacting Basal GFR
Date of Completion: May 7, 2026
Disclosure Updated Date: May 7, 2026

ASN
American Society of Nephrology
JASN
Journal of the American Society of Nephrology
KIDNEY360
Accessing Our World From Every Angle
CJASN
Clinical Journal of the American Society of Nephrology

ASN Journal Disclosure Form

As per ASN journal policy, I have disclosed any financial relationships or commitments I have held in the past 36 months as included below. I have listed my Current Employer below to indicate there is a relationship requiring disclosure. If no relationship exists, my Current Employer is not listed.

E. Rajadhyaksha reports the following:
Employer: Indiana University School of Medicine

I understand that the information above will be published within the journal article, if accepted, and that failure to comply and/or to accurately and completely report the potential financial conflicts of interest could lead to the following: 1) Prior to publication, article rejection, or 2) Post-publication, sanctions ranging from, but not limited to, issuing a correction, reporting the inaccurate information to the authors' institution, banning authors from submitting work to ASN journals for varying lengths of time, and/or retraction of the published work.

Name: Evan Ajit Rajadhyaksha
Manuscript ID: JASN-2026-000163R1
Manuscript Title: Recurrent Pyelonephritis Causes Kidney Fibrosis and Impairs Renal Functional Reserve Without Impacting Basal GFR
Date of Completion: May 6, 2026
Disclosure Updated Date: May 6, 2026

ASN Journal Disclosure Form

As per ASN journal policy, I have disclosed any financial relationships or commitments I have held in the past 36 months as included below. I have listed my Current Employer below to indicate there is a relationship requiring disclosure. If no relationship exists, my Current Employer is not listed.

V. Saxena reports the following:
Employer: Indiana University

I understand that the information above will be published within the journal article, if accepted, and that failure to comply and/or to accurately and completely report the potential financial conflicts of interest could lead to the following: 1) Prior to publication, article rejection, or 2) Post-publication, sanctions ranging from, but not limited to, issuing a correction, reporting the inaccurate information to the authors' institution, banning authors from submitting work to ASN journals for varying lengths of time, and/or retraction of the published work.

Name: Vijay Saxena
Manuscript ID: JASN-2026-000163R1
Manuscript Title: Recurrent Pyelonephritis Causes Kidney Fibrosis and Impairs Renal Functional Reserve Without Impacting Basal GFR
Date of Completion: May 7, 2026
Disclosure Updated Date: May 7, 2026

ASN
American Society of Nephrology
JASN
Journal of the American Society of Nephrology
KIDNEY360
Accessing Our World From Every Angle
CJASN
Clinical Journal of the American Society of Nephrology

ASN Journal Disclosure Form

As per ASN journal policy, I have disclosed any financial relationships or commitments I have held in the past 36 months as included below. I have listed my Current Employer below to indicate there is a relationship requiring disclosure. If no relationship exists, my Current Employer is not listed.

A. Schwaderer reports the following:
Employer: Indiana University

I understand that the information above will be published within the journal article, if accepted, and that failure to comply and/or to accurately and completely report the potential financial conflicts of interest could lead to the following: 1) Prior to publication, article rejection, or 2) Post-publication, sanctions ranging from, but not limited to, issuing a correction, reporting the inaccurate information to the authors' institution, banning authors from submitting work to ASN journals for varying lengths of time, and/or retraction of the published work.

Name: Andrew L. Schwaderer
Manuscript ID: JASN-2026-000163R1
Manuscript Title: Recurrent Pyelonephritis Causes Kidney Fibrosis and Impairs Renal Functional Reserve Without Impacting Basal GFR
Date of Completion: May 11, 2026
Disclosure Updated Date: May 11, 2026

ASN
American Society of Nephrology
JASN
Journal of the American Society of Nephrology
KIDNEY360
Accessing Our World From Every Angle
CJASN
Clinical Journal of the American Society of Nephrology

ASN Journal Disclosure Form

As per ASN journal policy, I have disclosed any financial relationships or commitments I have held in the past 36 months as included below. I have listed my Current Employer below to indicate there is a relationship requiring disclosure. If no relationship exists, my Current Employer is not listed.

A. Shaw reports the following:
Employer: Community Health Network

I understand that the information above will be published within the journal article, if accepted, and that failure to comply and/or to accurately and completely report the potential financial conflicts of interest could lead to the following: 1) Prior to publication, article rejection, or 2) Post-publication, sanctions ranging from, but not limited to, issuing a correction, reporting the inaccurate information to the authors' institution, banning authors from submitting work to ASN journals for varying lengths of time, and/or retraction of the published work.

Name: Alleé M Shaw
Manuscript ID: JASN-2026-000163R1
Manuscript Title: Recurrent Pyelonephritis Causes Kidney Fibrosis and Impairs Renal Functional Reserve Without Impacting Basal GFR
Date of Completion: May 13, 2026
Disclosure Updated Date: May 13, 2026

ASN
American Society of Nephrology
JASN
Journal of the American Society of Nephrology
KIDNEY360
Accessing Our World From Every Angle
CJASN
Clinical Journal of the American Society of Nephrology

ASN Journal Disclosure Form

As per ASN journal policy, I have disclosed any financial relationships or commitments I have held in the past 36 months as included below. I have listed my Current Employer below to indicate there is a relationship requiring disclosure. If no relationship exists, my Current Employer is not listed.

D. Soranno has nothing to disclose.

I understand that the information above will be published within the journal article, if accepted, and that failure to comply and/or to accurately and completely report the potential financial conflicts of interest could lead to the following: 1) Prior to publication, article rejection, or 2) Post-publication, sanctions ranging from, but not limited to, issuing a correction, reporting the inaccurate information to the authors' institution, banning authors from submitting work to ASN journals for varying lengths of time, and/or retraction of the published work.

Name: Danielle Elise Soranno
Manuscript ID: JASN-2026-000163R1
Manuscript Title: Recurrent Pyelonephritis Causes Kidney Fibrosis and Impairs Renal Functional Reserve Without Impacting Basal GFR
Date of Completion: May 7, 2026
Disclosure Updated Date: April 23, 2026
