# Supplementary material for: Recurrent Antibiotic-Treated Pyelonephritis Depletes Renal Functional Reserve Despite Preserved Basal GFR in a Reflux-Prone Mouse Model
Source: J Am Soc Nephrol. 2026 Jun 5;37(9):2027–9. doi: 10.1681/ASN.0000001166 (PMC13567891; doi:10.1681/ASN.0000001166)
Supplement: Supplementary file 2 [file jasn-37-2027-s002.pdf]

Supplemental Material

Supplemental Methods

Supplemental Figure 1. Experimental timeline.

Supplemental Methods

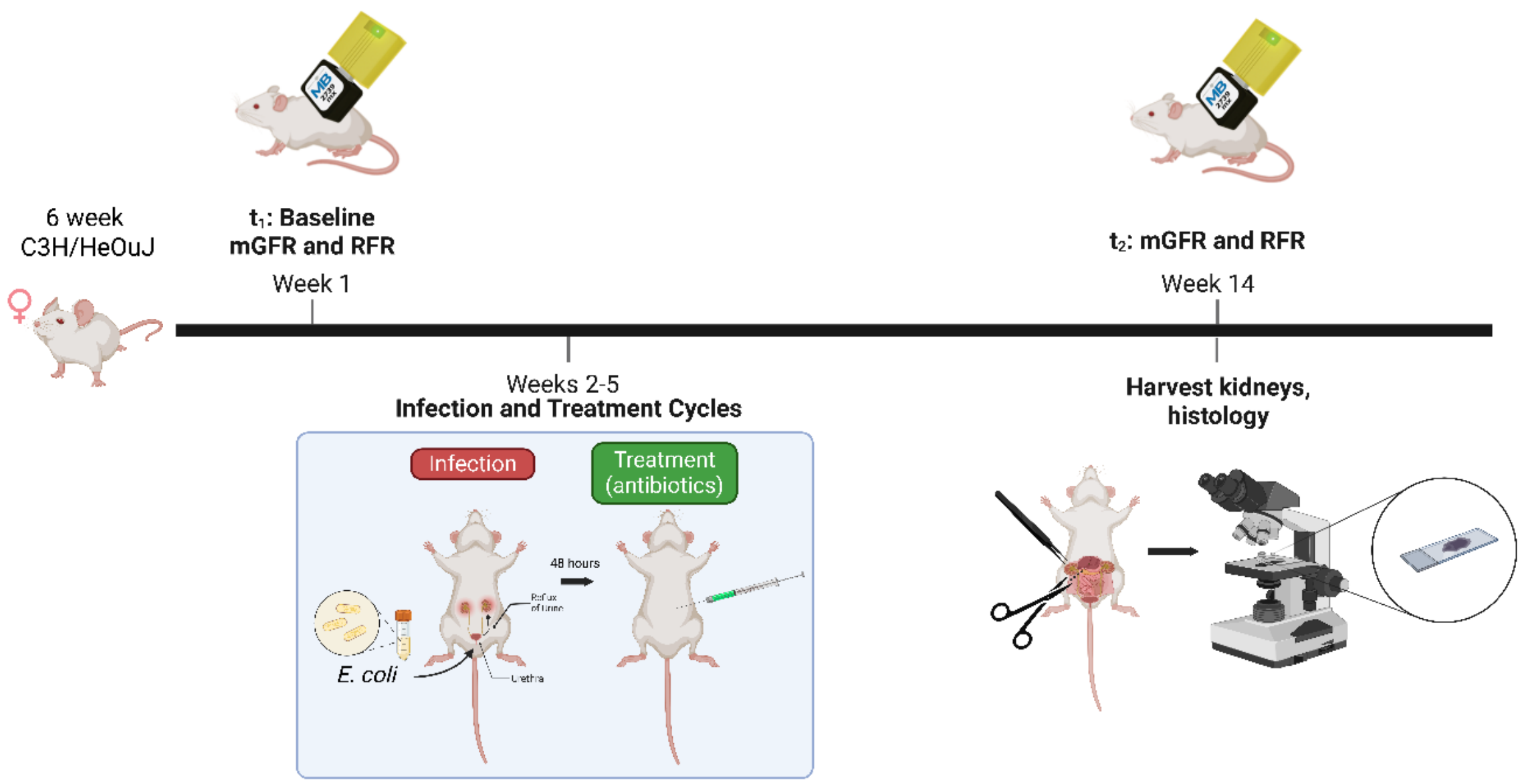

	t_1: Baseline mGFR and RFR	Infection and Treatment Cycles	t_2: mGFR and RFR	Kidney Histology
Control Group	✓	✗	✓	✓
Single Infection Group	✓	✓	✓	✓
Recurrent Infection Group	✓	✓ ✓ ✓ ✓	✓	✓

Supplemental Figure 1. Experimental timeline. *Created in BioRender. Rajadhyaksha, E. (2026) https://BioRender.com/ls19tm8*

Animals and Experimental Design

Female C3H/HeOuJ mice (Jackson Laboratory, Bar Harbor, Maine, USA; JAX stock #000635), were used for this experiment because this strain exhibits vesicoureteral reflux (VUR)[1] and consistently develops bilateral pyelonephritis following transurethral inoculation of uropathogenic *E. coli* (UPEC) into the bladder.[2] Animals were housed in an AAALAC-accredited facility. All procedures were approved by the Indiana University Institutional Animal Care and Use Committee, Protocol 23125, and adhered to the NIH Guide for the Care and Use of Laboratory Animals.

Cohort 1 Design and Induction of Pyelonephritis

Cohort 1 was designed to characterize histologic injury from pyelonephritis, and to determine if fibrosis severity correlates with number of infections. Six-week-old female C3H/HeOuJ mice (n=40) were acclimated for 1 week then randomized to receive zero (n=10), one (n=15), or four (n=15) bacterial inoculations and antibiotic treatment cycles. The noninfected control group was further divided into untreated (n=5) versus antibiotic-treated (n=5) to evaluate potential modifying effect from cephalosporin use.

Pyelonephritis was induced under brief inhalational anesthesia via transurethral inoculation with 10^8 CFU UPEC, strain CFT073. In clinical practice, parents are advised to seek UTI evaluation after ≥24 hours of fever.[3] Infected and antibiotic-treated mice received intraperitoneal cefpirome 40 mg/kg starting at 48 hours-post-infection (hpi), twice daily for 5 doses with each infection cycle.[4] The recurrent infection group underwent four infection and treatment cycles at 1-week intervals, versus one cycle for the single infection group. The antibiotic-only subset of the control group received cefpirome for four total cycles, on the same timeline as the recurrent infection mice, but neither control group underwent bacterial inoculation.

Measured GFR (mGFR) was an exploratory outcome in this cohort. Pre-intervention basal GFR was measured in a subset of 15 mice (5 each from control, single infection, and recurrent infection groups). Post-intervention GFR and euthanasia were 4 weeks following the final infection and treatment cycle for each group. Untreated controls were euthanized with the single infection group around 12 weeks of age, and antibiotic-treated controls were sacked with the recurrent infection group at 15 weeks of age.

Validation of Infection and Treatment Model

Susceptibility of *E. coli* strain CFT073 (GFP-producing) to cefpirome was tested using a cefpirome-enriched LB agar plate, and cefpirome successfully inhibited growth on the culture plate. An additional cohort of mice (n=12) was used to validate infection and clearance of the infection *in vivo*. Prior to infection, urine culture was negative for all mice. At 24 hpi, urine culture was positive for all mice, indicating urinary tract infection. Additionally, all kidneys (n=8) and bladders (n=4) showed robust growth of bacteria

through 10^7 dilution, reflecting $>10^7$ colony forming units/mL homogenized tissue and demonstrating 100% success rate in inducing infection in the kidney. Cefpirome was given BID from 48 through 96 hpi, and urine, kidney, and bladder bacterial burdens were reassessed at 7 days post-infection (dpi). At this timepoint, all mice had negative urine (n=8), kidney (n=16), and bladder (n=8) cultures, demonstrating 100% clearance of the infection with the reported antibiotic therapy.

Histology and Collagen Quantification

At euthanasia, kidneys were harvested, immersion-fixed in 4% paraformaldehyde for 24 hours, and transferred to 70% ethanol. Paraffin-embedded kidneys were sectioned at 5 µm. Sections were stained with Masson’s trichrome and imaged under brightfield microscopy using the ECHO Revolution microscope (ECHO, San Diego, CA, USA). Color deconvolution stain vectors were defined using Qupath 6.0 and applied to images in ImageJ 1.54. Total tissue area and blue, collagen-positive area were quantified using standardized thresholding and segmentation parameters on color-deconvoluted image of blue stain. Percent collagen area was calculated as (collagen-positive area / total tissue area) × 100.

GFR Measurement

GFR was measured using retroorbital fluorescein isothiocyanate (FITC)–sinistrin and a transdermal renal function monitor (Preclinical eGFR Monitoring System, MediBeacon, Mannheim, Germany), per the manufacturer’s instructions and aligned with established protocols.[5] Transdermal FITC–sinistrin clearance has been validated against plasma clearance methods and enables serial, longitudinal GFR assessment in small animals.[6,7] Measurements were obtained in conscious, freely moving mice placed temporarily in individual cages with access to food and water. MediBeacon software was used to derive mGFR in mL/min/100g body weight, which was then converted to mL/min/1.73 m^2 using a species-specific conversion factor (Km = 3 m^2/kg for mice).[8] If a technical complication occurred during injection (e.g., injection into the globe or nasal cavity), the measurement was discarded and repeated within 1 week. The frequency of repeated measurements was ~10% and did not differ among groups.

Cohort 2 Design and Renal Functional Reserve Protocol

Cohort 2 (n=22) was designed to evaluate functional outcomes and used “stimulated” GFR measurements to assess the functional reserve of the kidney. Mice were acquired at 5 weeks, acclimated for 1 week, and then assigned to receive zero (n=6), one (n=8), or four (n=8) infection–treatment cycles. This cohort was designed to have approximately 80% power to detect an absolute change of 20% in RFR between baseline and end of study values within groups, using a paired t-test with two-sided α = 0.05.

Basal mGFR and stimulated mGFR were quantified in the same animal, and renal functional reserve was calculated as the difference between stimulated and basal mGFR.[6,7] Stimulated GFR measurements were performed 1 hour after protein loading with 5 g/kg rehydrated meat extract (Millipore #70164) via oral gavage, adapted from an RFR model used in rats,[9] and compared to unstimulated (basal) GFR measurements taken the day prior. RFR was calculated as RFR = $100^*(GFR_{stimulated} – GFR_{unstimulated}) /GFR_{unstimulated}$ and is expressed as a percent. Each mouse underwent RFR testing at "t_1" = "baseline," prior to interventions, and "t_2" = "end of study," at 18-20 weeks of age and corresponding to at least 8 weeks following the recurrent pyelonephritis group's final infection-treatment cycle. Technical repeats were performed as described in "GFR measurement," above. All mice were euthanized following t_2 RFR testing, and histology was assessed using the same methods as for cohort 1.

Statistical Analysis

Normally distributed continuous data are reported as mean ± SD and non-normally distributed data are reported as median [IQR] unless otherwise indicated. We defaulted to nonparametric testing for group comparisons if any single group did not pass normality testing. Collagen quantification by trichrome stain was analyzed using Kruskal-Wallis test with Dunn's post-hoc multiple comparisons. mGFR and RFR values were compared between groups using Welch's ANOVA, and within groups using paired t test. A two-sided p<0.05 was considered statistically significant. All analyses were performed using GraphPad Prism version 10.6.1 (GraphPad Software, San Diego, CA).

References for Supplemental Methods

1 Hopkins, W., Gendron-Fitzpatrick, A., McCarthy, D. O., Haine, J. E. & Uehling, D. T. Lipopolysaccharide-responder and nonresponder C3H mouse strains are equally susceptible to an induced Escherichia coli urinary tract infection. *Infect Immun* **64**, 1369-1372 (1996). https://doi.org/10.1128/iai.64.4.1369-1372.1996

2 Li, B. *et al*. Inflammation drives renal scarring in experimental pyelonephritis. *Am J Physiol Renal Physiol* **312**, F43-F53 (2017). https://doi.org/10.1152/ajprenal.00471.2016

3 Shaikh, N. *et al*. Does this child have a urinary tract infection? *JAMA* **298**, 2895-2904 (2007). https://doi.org/10.1001/jama.298.24.2895

4 Arai, S. & Hayashi, S. Therapeutic effects of cefpirome (HR 810) on experimental mixed infections with Enterococcus faecalis and Escherichia coli in mice. *Infection* **18**, 186-190 (1990). https://doi.org/10.1007/BF01642112

5 Soranno, D. E. *et al*. Acute Kidney Injury Results in Long-Term Diastolic Dysfunction That Is Prevented by Histone Deacetylase Inhibition. *JACC Basic Transl Sci* **6**, 119-133 (2021). https://doi.org/10.1016/j.jacbts.2020.11.013

6 Schreiber, A. *et al.* Transcutaneous measurement of renal function in conscious mice. *Am J Physiol Renal Physiol* **303**, F783-788 (2012). https://doi.org/10.1152/ajprenal.00279.2012

7 Schock-Kusch, D. *et al.* Transcutaneous measurement of glomerular filtration rate using FITC-sinistrin in rats. *Nephrol Dial Transplant* **24**, 2997-3001 (2009). https://doi.org/10.1093/ndt/gfp225

8 Nair, A. B. & Jacob, S. A simple practice guide for dose conversion between animals and human. *J Basic Clin Pharm* **7**, 27-31 (2016). https://doi.org/10.4103/0976-0105.177703

9 Taylor, K. M. *et al.* Kidney functional reserve and damage biomarkers in subclinical chronic kidney disease and acute kidney injury. *Am J Physiol Renal Physiol* **325**, F888-F898 (2023). https://doi.org/10.1152/ajprenal.00133.2023
